# DETERMINANTS OF NEUROCOGNITIVE IMPAIRMENT AND DEMENTIA IN ENGLAND AND JAPAN

**DOI:** 10.1093/geroni/igz038.682

**Published:** 2019-11-08

**Authors:** Dorina Cadar, Kokoro Shirai

**Affiliations:** 1 University College London, London, United Kingdom; 2 Graduate School of Medicine Osaka University, Suita, Osaka, Japan

## Abstract

Dementia is one of the major contributors to disability and dependency amongst the elderly populations and a significant public health concern. Even though the prevalence of dementia in the UK is rising due to higher numbers of people surviving into older ages, recent evidence suggests that the UK is experiencing a decline in dementia incidence. By contrast, Japan has witnessed a different trend, with increases in both incidence and prevalence. This difference could be related to diagnostic practices within each country, or to the cultural variability in the risk and protective factors driving these emerging forecasts that remain fundamentally different between the UK and Japan. Research in this field has been dominated by clinical studies of dementia mostly conducted in the UK and US, and the current evidence lacks reliable national data on dementia incidence. Socioeconomic inequalities and social determinants of neurocognitive health and dementia risk in two longitudinal studies of ageing: the English Longitudinal Study of Ageing (ELSA) from the UK and Japan Gerontological Evaluation Study of Aging (JAGES). These studies are ideally placed for addressing pivotal research questions in gerontology: 1. What are the biopsychosocial determinants of cognitive impairment and dementia in England and Japan? 2. What are the potential exploratory mechanisms related to the divergent trends in dementia incidence observed in England and Japan? 3. What are the critical differences between the social determinants of dementia in England and Japan?

